# Improved prediction of anti-angiogenic peptides based on machine learning models and comprehensive features from peptide sequences

**DOI:** 10.1038/s41598-024-65062-9

**Published:** 2024-06-22

**Authors:** Yun-Chen Lee, Jen-Chieh Yu, Kuan Ni, Yu-Chuan Lin, Ching-Tai Chen

**Affiliations:** 1 Department of Computer Science and Information Engineering, Asia University, Taichung, 41354 Taiwan; 2 Department of Bioinformatics and Medical Engineering, Asia University, Taichung, 41354 Taiwan; 3 Graduate Institute of Genomics and Bioinformatics, National Chung Hsing University, Taichung, 40227 Taiwan; 4 Center for Precision Health Research, Asia University, Taichung, 41354 Taiwan

**Keywords:** Computational models, Machine learning

## Abstract

Angiogenesis is a key process for the proliferation and metastatic spread of cancer cells. Anti-angiogenic peptides (AAPs), with the capability of inhibiting angiogenesis, are promising candidates in cancer treatment. We propose AAPL, a sequence-based predictor to identify AAPs with machine learning models of improved prediction accuracy. Each peptide sequence was transformed to a vector of 4335 numeric values according to 58 different feature types, followed by a heuristic algorithm for feature selection. Next, the hyperparameters of six machine learning models were optimized with respect to the feature subset. We considered two datasets, one with entire peptide sequences and the other with 15 amino acids from peptide N-termini. AAPL achieved Matthew’s correlation coefficients of 0.671 and 0.756 for independent tests based on the two datasets, respectively, outperforming existing predictors by a range of 5.3% to 24.6%. Further analyses show that AAPL yields higher prediction accuracy for peptides with more hydrophobic residues, and fewer hydrophilic and charged residues. The source code of AAPL is available at https://github.com/yunzheng2002/Anti-angiogenic.

## Introduction

Cancer encompasses a group of diseases characterized by the unregulated growth of abnormal cells, with the capacity to infiltrate adjacent healthy tissues and disseminate via the circulatory or lymphatic system, a phenomenon referred to as metastasis^1^. Angiogenesis is a process by which new blood vessels are formed and it is seen as one of the key processes for the proliferation and metastatic spread of cancer cells. It promotes the circulation of oxygenated blood, supplies nutrients, and removes waste products from the body^2^. Most solid tumors, such as those found in the lung, breast, colon, prostate, and many other organs, rely heavily on angiogenesis for their growth and progression. Take lung cancer for example, it accounted for 2.5 million new cases (12.4% of all cancers) of all newly diagnosed cancers in 2022. In the same year, an estimated 1.8 million deaths (18.7%) from cancer were attributed to lung cancer, making it a leading cause of cancer-related mortality^3^. Currently, the modulation and suppression of angiogenesis represent forefront investigations in the field of cancer therapy^4,5^, with significant implications for the treatment of other diseases dependent on angiogenesis such as blindness, rheumatoid arthritis, and psoriasis^6–8^.

Several peptides, originating from diverse proteins, possess the capability to inhibit angiogenesis^5,9–12^. However, experimental techniques employed in the discovery and optimization of anti-angiogenic peptides (AAPs) have been markedly time-consuming, costly, and arduous, prompting the need for more efficient and effective approaches. With the growing number of AAPs available in databases, identifying potential AAPs based on computational models is highly desirable.

To date, several computational methods have been developed for the identification of AAP. AntiAngioPred^13^ considers peptide features of amino acid composition (AAC) and dipeptide composition (DPC) with seven machine learning (ML) models to predict AAP, among which support vector machines (SVM) with the feature of AAC yields the best prediction performance. Blanco et al*.*^14^ take AAC, DPC, and tripeptide composition as feature types, filter irrelevant numerical features using T-test, and conduct experiments using four ML models, among which the generalized linear model generates the highest prediction accuracy. TargetAntiAngio uses features of AAC, pseudo amino acid composition (PseAAC), and amphiphilic pseudo amino acid composition with a random forest model to predict AAPs. AntAngioCOOL^15^ uses PseAAC, several composition-based features, physicochemical profiles of 5 amino acids from N-termini and C-termini, and atomic profiles as features and employs 227 classifiers. Among all the classifiers, the three models that achieve the highest sensitivity, specificity, and accuracy, respectively, are included in their software package. AAPred-CNN is a deep learning model that adopts multiple convolution channels to extract local features of input sequences to identify AAP. It is reported to outperform existing methods in all evaluation metrics.

While existing studies have shown some advancements, a majority of them face limitations due to their choice of feature types and the lack of a systematic approach to determine suitable feature subsets and optimize ML models. More importantly, there is still room to improve the prediction accuracy. In this study, we present AAPL, a sequence-based predictor of AAP which considers a comprehensive set of features from peptide sequences. We considered 58 different feature types, producing a total of 4335 numeric values for each sequence. Next, feature ranking and a heuristic feature selection algorithm were applied to determine the best feature subset for ML. The hyperparameters of six different ML models were then optimized with respect to the selected feature subset. The resulting models were used to conduct cross validation and independent tests for benchmark analysis. The evaluation results, derived from two independent test datasets, indicate that AAPL significantly outperforms current methods in terms of prediction accuracy. The situation affirms that AAPL, by leveraging a comprehensive array of features extracted from peptide sequences, serves as an efficient and precise approach for the identification of AAP.

## Materials and methods

### Datasets

Two datasets used in several studies^8,14–16^ were downloaded from the AntiAngioPred^13^ web server. The first dataset consisted of 133 AAPs with experimental validation and 135 non-AAPs randomly selected from SwissProt^17^. To minimize bias, the dataset included only pairs of peptides with a sequence identity less than 70%, and the lengths of both AAPs and non-AAPs were similarly distributed. An 80% split of the dataset was applied, resulting in 105 positive peptides and 107 negative peptides, termed S212, for model development and cross validation. The other 20% of sequences, termed S56, consisted of 28 AAPs and 28 non-AAPs for independent test.

In addition to the datasets of full peptide length, we also considered terminus datasets, i.e., datasets consisting of the first 15 amino acids from the N-termini of peptides^8,13,16^. Two terminus datasets were obtained from TargetAntiAngio^16^. The one for model development and cross validation, termed NT-S160, consisted of 80 AAPs and 80 non-AAPs. The one for independent test, termed NT-S40, consisted of 19 AAPs and 21 non-AAPs.

### Workflow of AAPL

**Figure 1:**
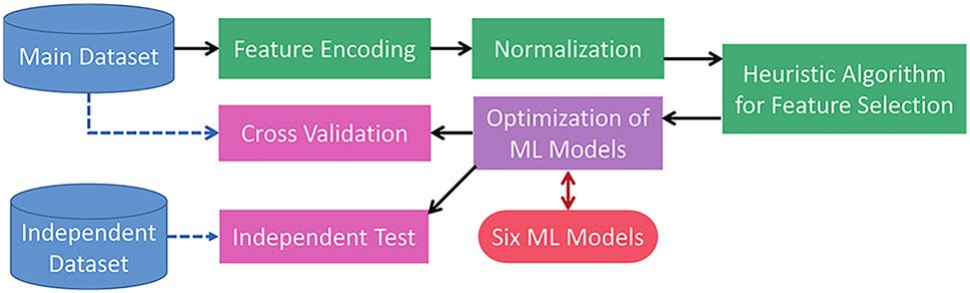
Workflow of AAPL.

Figure 1 illustrates the workflow of AAPL. The main dataset (consisting of 80% of sequences) was used for feature engineering, including procedures of feature encoding, normalization, and a heuristic algorithm for feature selection. The selected feature subset was then used to tune the hyperparameters of ML models using a Bayesian optimization algorithm. Next, the ML models were employed for cross validation with the main dataset and independent test with an independent dataset. The entire workflow is detailed in the following sections.

### Feature encoding

We transformed amino acid sequences into numerical representations by a variety of compositional and physicochemical properties of peptides. A total of 58 feature types were considered, including peptide length, AAC, DPC, grouped amino acid composition (GAAC)^18^*,* grouped di-peptide composition (GDPC)^19^, composition of k-spaced amino acid pairs (CKSAAP)^20^, amphiphilic pseudo amino acid composition (APAAC)^21–23^, composition of k-spaced amino acid group pairs (CKSAAGP)^24,25^, Shannon entropy at protein level (SEP)^26,27^, Shannon entropy at residue level (SER)^26,27^, Composition-Transition-Distribution (CTD)^28^, conjoint triad (CTriad)^29^, dipeptide deviation from expected mean (DDE)^30^ etc. The entire list of 58 feature types and the size of each of them (number of numeric values) are listed in Supplementary Table S1. Each sequence was associated with a feature vector of 4335 entries.

### Normalization

Robust normalization was applied to compositional and physicochemical features with *RobustScaler* from the *Scikit-learn* package^31^ of Python. RobustScaler converts a feature value *x*_*i*_ to *y*_*i*_ using the following equation.

1 $$y_{i} = \frac{{x_{i} - Median\left( X \right)}}{Q3\left( X \right) - Q1\left( X \right)},$$

in which *Q3*(*X*) and *Q1*(*X*) stand for the 3rd quartile and the 1st quartile of feature *X*. The scaled values of each feature have a median of zero and an interquartile range of one across all sequences.

### Machine learning methods

Six ML models were considered in this study, including support vector machines (SVM)^32^, linear discriminant analysis (LDA)^33^, random forest (RF)^34^, extremely randomized trees (ET)^35^, light gradient boosting machine (LightGBM)^36^, and categorical boosting (CB)^37^. SVM is a type of kernel-based learning algorithm that transforms the data into a higher-dimensional space, and then searches for a hyperplane in the space that maximizes the margin between the two classes. We used the radial basis function as the kernel for SVM. LDA aims to find linear combinations of features that maximize the separation between different classes in the data while minimizing the variance within each class. RF constructs a multitude of decision trees, trained on random subsets of the data and random subsets of features. The predictions of the individual trees are then averaged to produce the final prediction of RF. ET is similar to RF, but differs in two key ways: (1) ET splits nodes on all features instead of a random subset of features, and (2) ET builds each tree on the entire training set, rather than a random subset of the training set. LightGBM and CB are two different implementations of gradient boosting algorithm, which constructs a sequence of weak learners, each trained to minimize the residual error of the previous weak learner. The final prediction of the gradient boosting model is a weighted average of the predictions of the individual weak learners.

### Heuristic algorithm for feature selection

Given *n* features, the potential number of feature subsets is *2*^*n*^. Exhaustively evaluating all possible combinations to find the optimal subset is computationally infeasible due to the fact that *n* is 4335 in this study. A heuristic algorithm for feature selection is therefore proposed. First, the Boruta program^38^, a wrapper approach utilizing a random forest algorithm for feature ranking, was employed to analyze all features. The algorithm progressively eliminates features that are statistically less relevant than randomized features, thereby generating a prioritized list of features based on their importance. For a given dataset, the ranked feature list is expressed as an ordered set *F* = {*F*_*1*_, *F*_*2*_, … *F*_*4335*_}, sorted in descending order according to feature importance. Next, a heuristic approach was applied to determine the feature subset for the dataset. The top *N* feature subset is defined as *FS*_*N*_ = {*F*_*1*_, *F*_*2*_, …*F*_*N*_}. Iterative five-fold cross validation runs using *FS*_*N*_ was performed based on SVM, LDA, RF, ET, LightGBM, and CB with their default hyperparameters (based on the settings of Scikit-learn package). The experiments were carried out via PyCaret^39^ and the results were evaluated with MCC. Let *MCC*_*N*_^*i*^ be the MCC for ML model *i* using *FS*_*N*_, The best MCC based on *FS*_*N*_ is defined as

2 $${Best\_MCC}_{N}=\underset{i}{\text{max}}({MCC}_{N}^{i})$$

where *i* corresponds to any of the six ML models (SVM, LDA, RF, ET, LightGBM, and CB). The process was repeated iteratively, with* N* ranging from 50 to 200 in increments of 10. The best feature subset (BFS) is defined as

3 $$BFS={FS}_{j}, where {Best\_MCC}_{j}=\underset{k}{\text{max}}{(Best\_MCC}_{k})$$

The purpose of feature selection is to determine the best feature subset for the given dataset. Using default hyperparameters of the six different ML models in the task may circumvent the lengthy process of ML model optimization. Moreover, using ML models of different rationale has the advantage that the selected feature subsets are not biased towards a particular model. The heuristic algorithm was applied to S212 and NT-S160, yielding two feature subsets.

### Optimization of machine learning models

After the feature subset was determined, hyperparameters of ML models were optimized on S212 and NT-S160 with Optuna^40^, an automatic hyperparameter optimization package for sampling search space and pruning unpromising trials using a Bayesian model. We employed the tree-structured Parzen estimator algorithm^41^ as the search algorithm of Optuna due to its superior ability to converge quickly to the global optimum compared to randomized search, while also demanding less computing time than grid search. After the optimized hyperparameters were obtained for each model, ten-fold cross validation was performed to evaluate each predictor.

To perform an independent test, we carried out an additional training phase utilizing the optimized parameters of each model on the complete S212 or NT-S160 dataset. The resulting models, benefiting from an additional 10% of training data compared to the models obtained from cross validation, were used to conduct independent tests based on S56 and NT-S40.

### Evaluation metrics

For benchmark comparison, prediction results were evaluated with accuracy, precision, recall (or sensitivity), F1-score, and MCC (Matthew’s Correlation Coefficient) defined as follows:

4 $$\begin{array}{*{20}c} {{\text{Accuracy (Acc) = }}\frac{{\text{TP + TN}}}{{\text{TP + TN + FP + FN}}}} \\ \end{array}$$

5 $$\begin{array}{*{20}c} {{\text{Precision (Pre) = }}\frac{{{\text{TP}}}}{{\text{TP + FP}}}} \\ \end{array}$$

6 $$\begin{array}{*{20}c} {{\text{Recall (Rec) = Sensitivity = }}\frac{{{\text{TP}}}}{{\text{TP + FN}}}} \\ \end{array}$$

7 $$\begin{array}{*{20}c} {{\text{Specificity (Sp)}} = \frac{{{\text{TN}}}}{{\text{TN + FP}}}} \\ \end{array}$$

8 $${\text{MCC}} = \frac{{{\text{TP}} \times {\text{TN}} -{\text{FP}} \times {\text{FN}}}}{{\sqrt {({\text{TP }} + {\text{ FP}})({\text{TP }} + {\text{ FN}})({\text{TN }} + {\text{ FP}})({\text{TN }} + {\text{ FN}})} }}$$

where TP, TN, FP, and FN denote the counts of true positives, true negatives, false positives, and false negatives, respectively. Accuracy, precision, recall, and specificity are on a scale from 0 to 1, where a higher value reflects better predictive performance. MCC spans from − 1 to 1, signifying entirely negative and entirely positive correlations, respectively, with an MCC of 0 indicating a random correlation. In addition to the above metrics, AUC (Area Under the receiver operating characteristic Curve)^42^, a non-parametric and threshold independent measure, was also included for evaluation. An AUC value of 1 indicates a perfect model, while an AUC value of 0.5 indicates a random guesser.

## Results and discussions

### Amino acid and dipeptide composition analyses

The AAC analysis of AAPs and non-AAPs for S212 is illustrated in Fig. S1. The analysis reveals that residues of C, P, R, S, and W are predominantly found in AAPs, whereas residues of A, E, I, L, and V are more commonly present in non-AAPs. The DPC analysis for S212 is shown in Fig. S2. Certain dipeptides like CG, CN, CS, HG, HH, SP, and SC are predominant in AAPs, while dipeptides such as AA, EL, EV, IA, and NK are more common in non-AAPs. The analyses show that the presence of certain amino acids and their synergistic interactions play a pivotal role in modulating the angiogenic properties of peptides.

### Selected feature subsets

**Figure 2:**
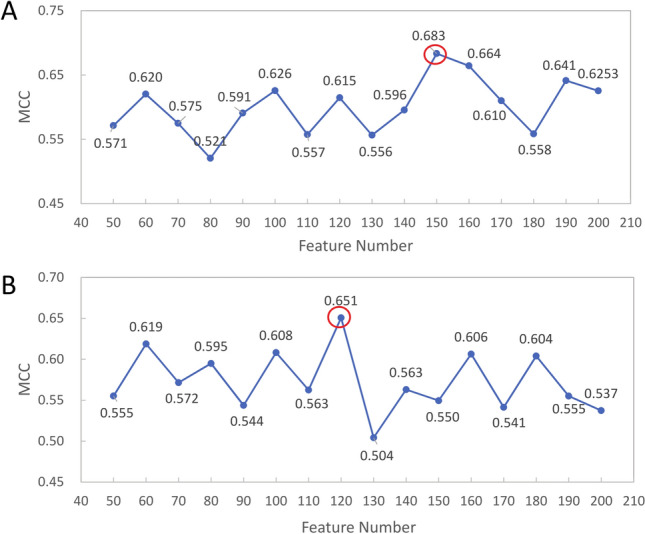
MCCs f the best performing ML models based on different feature numbers on (**A**) S212 and (**B**) NT-S160. The highest MCC in each panel is circled in red.

Cross validation results of S212 and NT-S160 based on different feature numbers were evaluated with MCC, and the results are shown in Fig. 2. It can be seen the best feature numbers of S212 and NT-S160 are 150 and 120, respectively. Using the two selected feature numbers, the best MCC achieved by the ML models are 0.683 and 0.651 for S212 and NT-S160, respectively. The entire lists of 150 features for S212 and 120 features for NT-S160 are shown in Supplementary Tables S2 and S3, respectively. The Gini importance produced by the random forest model is also listed for comparison. The features of S212 and NT-S160 are associated with 34 and 32 feature types, respectively, among which 28 feature types are in common. Table 1 lists the top 5 most frequent feature types for S212 and NT-S160. All of them are among the 28 common feature types, despite their differences in the number of selected features for each feature type. The entire lists of feature types for S212 and NT-S160 are shown in Table S4.

**Table 1:** The selected numbers and sizes of the top 5 most frequent feature types from the selected feature subset using S212 and NT-S160.

S212			NT-S160		
Feature type	Selected Num	Size	Feature type	Selected num	Size
CKSAAGP	19	150	Z5	21	75
Cougar	15	30	Cougar	16	30
Z5	15	75	Ez	14	30
Ez	13	30	Z3	9	30
CTDC	8	39	ABHPRK	6	105

### Feature exploration and biological relevance

The selected feature subset of S212 (Supplementary Table S2) consists of features regarding to certain amino acids, including Ala, Cys, Ser, Trp, Leu, and Phe. For example, amino acid composition (AAC) for Ala, Cys, and Ser, distance distribution of residues^43^ (DDR) for Ala, Cys, and Trp, and Shannon entropy at residue level (SER) for Ala, Cys, and Ser. This is in good agreement to the propensity shown in Supplementary Fig. S1, namely, Cys, Ser, and Trp are prevalent in AAPs, while Ala, Leu, and Phe are more common in non-AAPs. There is also a strong association between hydrophobicity and the majority of the selected features of CTD. The situation is consistent to the established fact that AAPs have a relatively high incidence of hydrophobic residues^44^. The presence of aliphatic residues appears to be a significant characteristic in CKSAAGP and GDPC. This observation aligns with the fact that aliphatic amino acids such as Ala, Ile, Leu, and Val tend to occur more frequently in AAPs, as indicated in Supplementary Fig. S1. In addition to the above, the feature subset also incorporates generalized feature types, such as Ez^45^, Z3^46^, and Z5^47^. Ez specifically characterizes the empirical residue-based potential for protein insertion in lipid membranes, a process governed by complex factors such as hydrophobic interactions, electrostatic forces, and hydrogen bonding. Meanwhile, Z3 and Z5 are multidimensional descriptors capturing hydrophobicity, charge, aromaticity, polarity, and other physicochemical properties critical to peptide behavior and function.

It can be observed from Supplementary Table S3 that compared to S212, NT-S160 exhibits a significantly smaller number of selected features across AAC, GDPC, and CKSAAGP, with only 1, 1, and 4 features selected, respectively, in contrast to 3, 4, and 19 features selected for S212. These feature types, which are derived from specific amino acids or their combinations, are likely influenced by the shorter sequence lengths present in NT-S160. Supplementary Table S3 reveals that hydrophobicity remains a predominant characteristic among the selected features of the CTD descriptor for NT-S160. Additionally, the feature types Ez, Z3, and Z5, which capture various physicochemical properties, still contribute significantly to the selected features, accounting for 14, 9, and 21 features, respectively.

### Benchmark results of cross validation

Table 2 shows the benchmark results of cross validation using the six different ML models on S212. It can be seen SVM, achieving an MCC of 0.642, outperforms the other models in all the evaluation measures. SVM outperforms CB, the second-best model, by 8.7%, 3.7%, and 4.2% in MCC, AUC, and accuracy, respectively. The precision of SVM is 0.840, representing an improvement over the other models by 3.6% to 9.9%. On the other hand, SVM generates a recall of 0.825, which is higher than other methods by a significant margin ranging from 7.6 to 11.5%, suggesting that recall plays a more critical role than precision in explaining the SVM’s substantial improvement in MCC on S212. Table 3 shows the benchmark results of cross validation on NT-S160. SVM achieves an MCC of 0.598 and generates the highest value in all evaluation measures. The MCCs of ET, RF, and CB are above 0.5. Similarly, SVM outperforms the other models by a range of 5.0% to 16.7% in recall, and by a range of 2.9% to 9.6% in precision, suggesting that the improved recall (or sensitivity) is the major reason why SVM yields the highest MCC on NT-S160. The ROC curves of the six ML models for S212 and NT-S160 are illustrated in Supplementary Fig. S3A,B, respectively.

**Table 2:** Benchmark results of cross validation on S212.

ML Model	Acc	Pre	Rec	Sp	AUC	MCC
SVM	**0.817**	**0.840**	**0.825**	**0.841**	**0.879**	**0.642**
CB	0.775	0.804	0.715	0.832	0.842	0.555
ET	0.769	0.801	0.710	0.822	0.830	0.546
LDA	0.769	0.794	0.738	0.813	0.845	0.544
RF	0.751	0.774	0.713	0.794	0.860	0.506
LightGBM	0.739	0.741	0.749	0.738	0.803	0.482

The best performance value is highlighted in bold for clarification.

**Table 3:** Benchmark results of cross validation on NT-S160.

ML Model	Acc	Pre	Rec	Sp	AUC	MCC
SVM	**0.797**	**0.843**	**0.767**	**0.863**	**0.873**	**0.598**
ET	0.766	0.814	0.717	0.838	0.880	0.556
RF	0.758	0.813	0.685	0.838	0.795	0.531
CB	0.749	0.824	0.635	**0.863**	0.817	0.518
LightGBM	0.711	0.747	0.635	0.788	0.794	0.428
LDA	0.711	0.759	0.600	0.813	0.777	0.428

The best performance value is highlighted in bold for clarification.

### Benchmark results of independent tests

Table 4 shows the benchmark results of S56 with the six ML models and three existing predictors, AAPred-CNN, TargetAntiAngio, and AntiAngioPred. It can be observed SVM yields the highest recall, accuracy, and MCC. SVM improves the MCC and recall of AAPred-CNN, the state-of-the-art method, by 5.3% and 17.8%, respectively, though AAPred-CNN generates the highest precision of 0.815. The AUC of SVM, 0.828, is comparable to the highest AUC of 0.830 produced by TargetAntiAngio. It can also be seen that AntiAngioPred yields the lowest MCC and recall, indicating a large number of false negatives. The situation is very likely caused by the fact that AntiAngioPred relies on a single feature type, amino acid composition, for prediction. The ROC curves of the six ML models for S56 are illustrated in Supplementary Fig. S4A.

**Table 4:** Benchmark results of independent test on S56.

Method	Acc	Pre	Rec	Sp	AUC	MCC
SVM	**0.821**	0.750	**0.964**	0.679	0.828	**0.671**
CB	0.768	0.727	0.857	0.679	0.763	0.544
RF	0.732	0.676	0.893	0.571	0.720	0.490
ET	0.732	0.686	0.857	0.607	0.738	0.480
LDA	0.714	0.750	0.643	0.786	0.700	0.433
LightGBM	0.714	0.688	0.786	0.643	0.736	0.433
AAPred-CNN	0.804	**0.815**	0.786	0.821	0.753	0.618
TargetAntiAngio	0.778	0.753	0.821	0.731	**0.830**	0.560
AntiAngioPred	0.696	–	0.536	**0.857**	–	0.410
AntAngioCOOL*	0.750	–	0.820	0.710	–	–

The best performance value is highlighted in bold for clarification.

*AntAngioCOOL package provides three different trained models. The evaluation measures for the model with the highest accuracy is used for comparison.

The evaluation results on NT-S40 are shown in Table 5. In consistence with previous studies, the overall MCCs for all methods are improved compared to the results of S56, the dataset of entire peptide sequences. SVM generates an MCC of 0.756, which is 5.9%, 19.6%, and 24.6% higher than AAPred-CNN, TargetAntiAngio, and AntiAngioPred, respectively. Notably, LightGBM, RF, ET, and CB also outperform the three existing methods in MCC. AAPred-CNN produces a precision and specificity of 1.000 but suffers from the second lowest recall of 0.650, indicating a large number of false negatives. In contrast, TargetAntiAngio produces the highest recall of 0.905 but suffers from the lowest precision among all the methods, indicating a large number of false positives. Nevertheless, the above benchmark comparisons demonstrate that our SVM model produces a significant improvement in MCC over existing methods. The ROC curves of the six ML models for NT-S40 are illustrated in Supplementary Fig. S4B.

**Table 5:** Benchmark results of independent test on NT-S40.

Method	Acc	Pre	Rec	Sp	AUC	MCC
SVM	**0.875**	0.938	0.789	0.952	**0.882**	**0.756**
LightGBM	**0.875**	0.850	0.895	0.857	0.847	0.751
RF	**0.875**	0.889	0.842	0.905	0.865	0.750
ET	0.850	0.810	0.895	0.810	0.872	0.704
CB	0.850	0.882	0.789	0.905	0.845	0.701
LDA	0.800	0.789	0.789	0.810	0.835	0.599
AAPred-CNN	0.825	**1.000**	0.650	**1.000**	0.765	0.697
TargetAntiAngio	0.775	0.690	**0.905**	0.632	0.840	0.560
AntiAngioPred	0.750	–	0.650	0.850	–	0.510

The best performance value is highlighted in bold for clarification.

**Figure 3:**
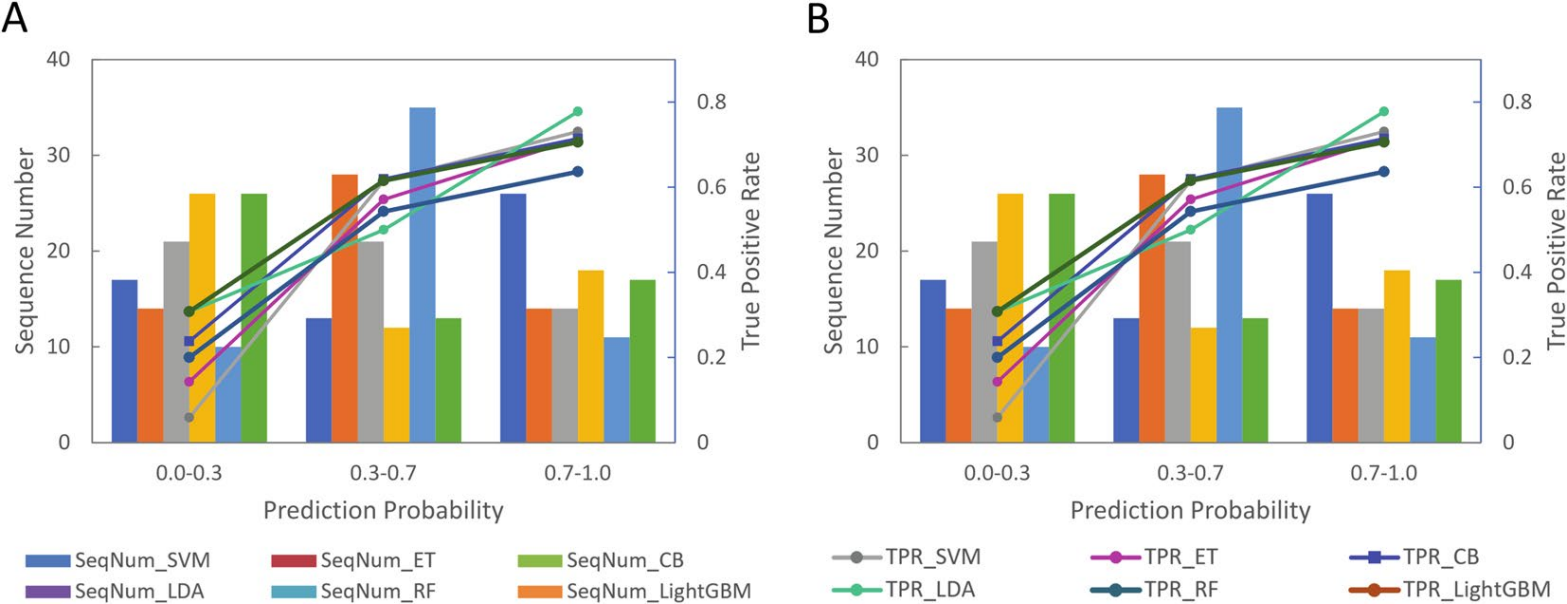
True positive rate (TPR) and sequence number (denoted as SeqNum in the figure) with respect to prediction probability of 6 ML models on (**A**) S56 and (**B**) NT-S40. Prediction probability for each sequence is obtained from the output of each machine learning model. True positive rate is calculated as the number of AAPs divided by the total number of sequences predicted within the range of the prediction probability.

We further analyzed the correlation between the prediction probability output for each model and the true positive rate (TPR), calculated by the number of actual AAPs divided by the total number of sequences predicted within the range of the prediction probability. As illustrated in Fig. 3A,B, all six ML models demonstrate strong positive correlations between the true positive rate and the prediction probability on both datasets. In other words, a higher prediction probability leads to a higher likelihood that the sequence is an actual AAP.

### Prediction accuracy with respect to peptide properties

**Figure 4:**
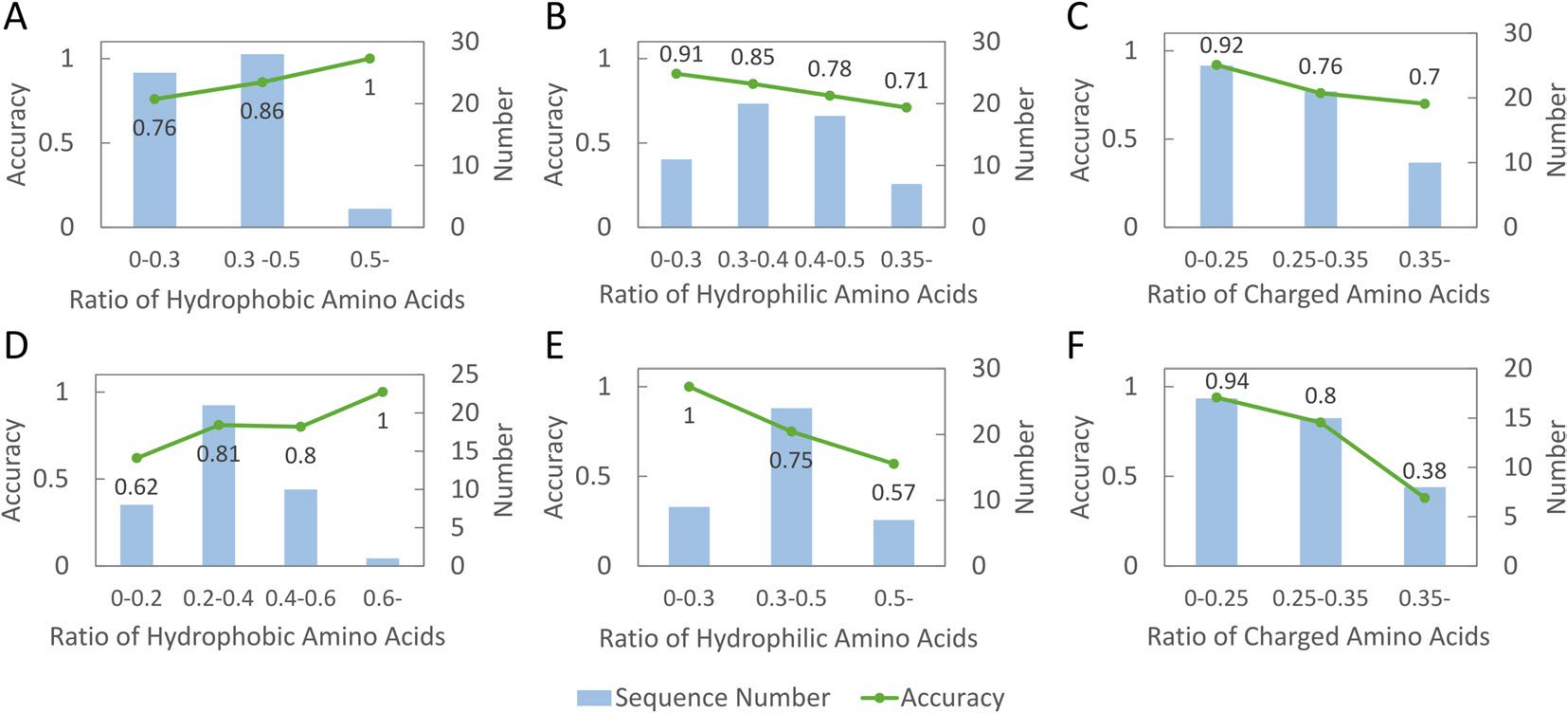
Analyses of prediction accuracy versus different properties of peptides from independent tests. Curves in panels **A**, **B**, and **C** represent mean accuracy of peptides with respect to peptides from S56 with different ratios of hydrophobic, hydrophilic, and charged residues. Curves in panels **D**, **E**, and **F** are defined analogously for peptides from NT-S40. Bars in each panel represent the number of peptides within the ratios specified by x-axis.

Prediction results from independent tests based on SVM, the most accurate model, were further analyzed with respect to three different peptide properties, namely, the ratios of hydrophobic, hydrophilic, and charged residues within a peptide. In this study, hydrophobic amino acids are V, I, L, M, F, W, and C; hydrophilic amino acids are R, N, D, E, Q, H, K, S, and T; charged amino acids are E, D, R, K, H. As illustrated in Fig. 4, the prediction accuracy is positively correlated with the ratio of hydrophobic residues within a peptide for S56 (Fig. 4A) and NT-S40 (Fig. 4D). On the other hand, the prediction accuracy is negatively correlated with the ratio of hydrophilic residues in a peptide (Fig. 4B for S56 and Fig. 4E for NT-S40) and the ratio of charged residues in a peptide (Fig. 4C for S56 and Fig. 4F for NT-S40). These results suggest peptides of more hydrophobic residues and fewer hydrophilic and charged residues are more accurately predicted. These are in agreement with prior studies which state that the hydrophobicity of a peptide is an important characteristic of AAP^16,44^. The analyses point towards potential areas for future refinement, specifically in improving predictions for peptides with fewer hydrophobic residues and a higher proportion of hydrophilic and charged residues.

### Efficacy of the selected feature subset

**Figure 5:**
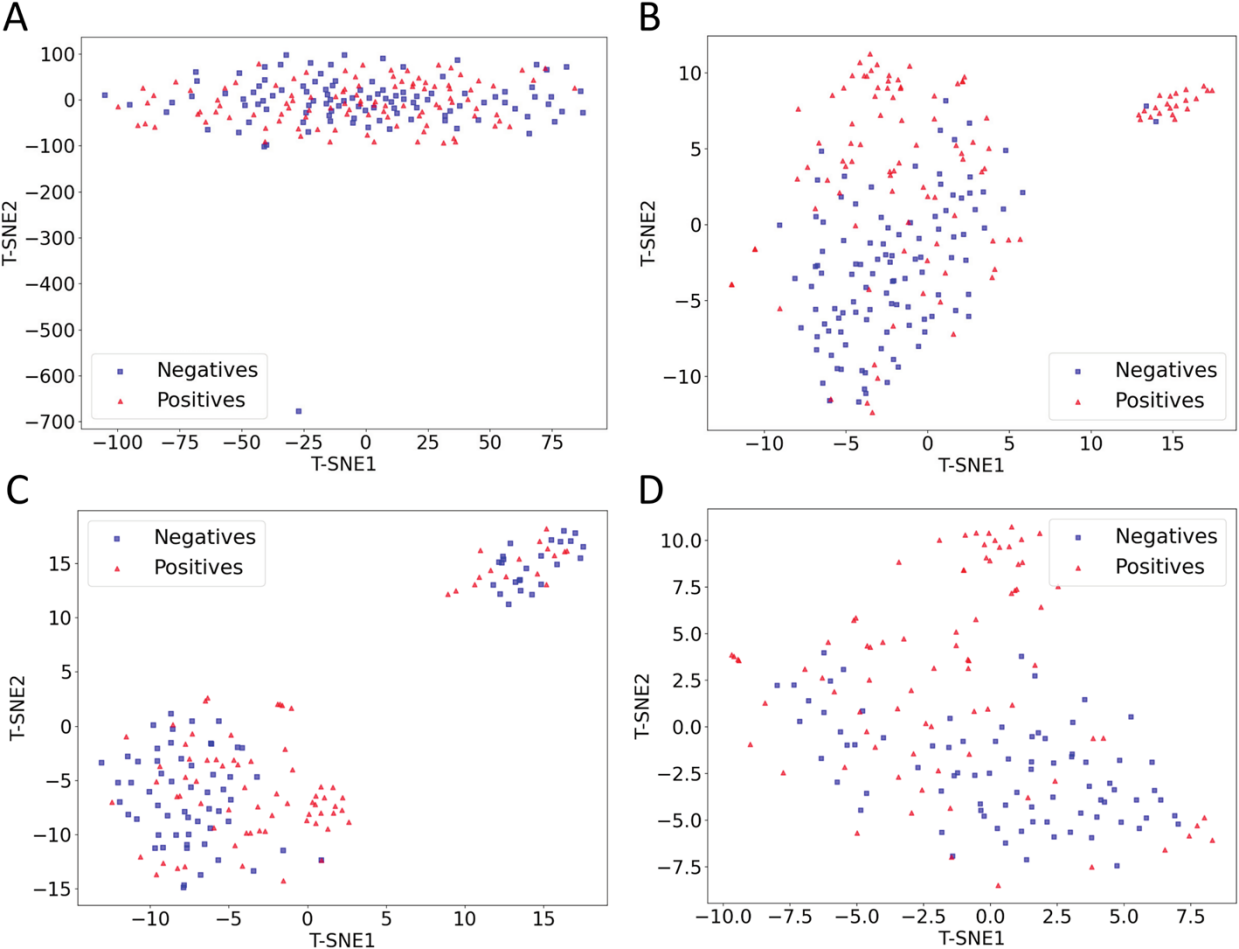
t-SNE distributions of (**A**) S212 using 4335 numeric features, (**B**) S212 using the 150 selected features, (**C**) NT-S160 using 4335 numeric features, and (**D**) NT-S160 using the 120 selected features. Negatives refer to non-AAPs and positives refer to AAPs.

In this study, each sequence is originally encoded with various compositional, physicochemical, and biological features, leading to a feature vector of 4335 numeric values. The selected feature subsets, consisting of 150 and 120 numeric values for S212 and NT-S160, respectively, play an important role in the enhanced prediction accuracy. To validate the efficacy of the feature subsets to the discrimination of TTCAs, we applied t-distributed stochastic neighbor embedding (t-SNE)^48,49^ to visualize data distributions on a two-dimensional plane. As illustrated in Fig. 5, the t-SNE distributions of negatives and positives have serious overlap using all 4335 numeric features for S212 (Fig. 5A) and NT-S160 (Fig. 5C). Conversely, the t-SNE distributions of negatives and positives are more separated using the selected feature subsets for S212 (Fig. 5B) and NT-S160 (Fig. 5D). Take Fig. 5B as an example, the positives are more concentrated in the upper left and upper right regions, while the negatives are more concentrated in the lower left region. The scenario reveals that the feature subsets for the two datasets are informative and beneficial to the improved prediction performances of ML models.

## Conclusion

In this study, we present AAPL, a sequence-based predictor for AAP prediction with improved prediction accuracy. Sequences were encoded with a comprehensive set of features, encompassing a total of 4335 numeric values based on 58 different feature types, followed by a feature ranking process that produces a ranked feature list according to feature importance. The best feature number was determined with a heuristic algorithm comprising iterative runs of cross validation using six different ML models. The feature subset corresponding to the highest MCC was then used for fine-tuning the hyperparameters of each ML model with a Bayesian optimization algorithm. The feature subset and the optimized ML models were then applied to conduct cross validation and independent tests. We considered two datasets, one consisting of full-length sequences and the other consisting of sequences of 15 residues from the peptide N-termini. The feature subsets of the former and the latter are composed of 150 and 120 numeric values, respectively. The independent test for the former shows that AAPL achieves an MCC of 0.671, which is 5.3%, 11.1%, and 26.1% higher than AAPred-CNN, TargetAntiAngio, and AntiAngioPred, respectively. The independent test for the latter shows that AAPL achieves an MCC of 0.756, which is 5.9%, 19.6%, and 24.6% higher than AAPred-CNN, TargetAntiAngio, and AntiAngioPred, respectively. Evaluation results reveal that AAPL’s higher recall, as opposed to precision, drives its superior prediction capability. Further analyses also demonstrate that the peptides of more hydrophobic residues, and fewer hydrophilic and charged residues are of higher prediction accuracy. The efficacy of the selected feature subsets was further validated with t-SNE plots. Compared to using the original 4335 numeric features, using the selected feature subsets results in greater separation between AAPs and non-AAPs in t-SNE plots, suggesting that the feature subsets are beneficial to ML models in improved prediction accuracy. Overall, the study shows that our machine learning-based approach achieves reasonably good prediction accuracy in identifying AAPs.

It is crucial to note that our models were developed using balanced datasets comprising equal proportions of AAPs and non-AAPs. This approach, albeit deviating from the anticipated real-world distribution, was adopted to facilitate model training and evaluation without introducing potential biases arising from class imbalance. Nonetheless, the work is considered an initial stage of development and future efforts can be made to explore the impact of class imbalance, thereby improving the robustness and generalizability of AAPL with more non-AAPs. In addition, future availability of experimentally validated AAPs could pave the way for deep learning-based prediction models.

### Supplementary Information

Supplementary Information 1.

Supplementary Tables.

## Data Availability

Source code of AAPL is available at https://github.com/yunzheng2002/Anti-angiogenic. Publicly available datasets analyzed in this study can be obtained from the website of AntiAngioPred at https://webs.iiitd.edu.in/raghava/antiangiopred/.
