# Supplementary material for: Improved prediction of anti-angiogenic peptides based on machine learning models and comprehensive features from peptide sequences
Source: Sci Rep. 2024 Jun 22;14:14387. doi: 10.1038/s41598-024-65062-9 (PMC11193773; doi:10.1038/s41598-024-65062-9)
Supplement: Supplementary file 1 — Supplementary Information 1. [file 41598_2024_65062_MOESM1_ESM.docx]

**Supplementary Material**

Improved Prediction of Anti-Angiogenic Peptides based on Machine Learning Models and Comprehensive Features from Peptide Sequences

Yun-Chen Lee^1,#^, Jen-Chieh Yu^2,#^, Kuan Ni^3^, Yu-Chuan Lin^2^, Ching-Tai Chen^2,4,*^

^1^Department of Computer Science and Information Engineering, Asia University, Taichung, Taiwan

^2^Department of Bioinformatics and Medical Engineering, Asia University, Taichung, Taiwan

^3^Graduate Institute of Genomics and Bioinformatics, National Chung Hsing University, Taichung 41354, Taiwan

^4^Center for Precision Health Research, Asia University, Taichung 41354, Taiwan

^#^The authors contributed equally to this work

*corresponding author. E-mail: [ctchen@asia.edu.tw](mailto:ctchen@asia.edu.tw), phone: +886-4-2332-3456 ext.1843


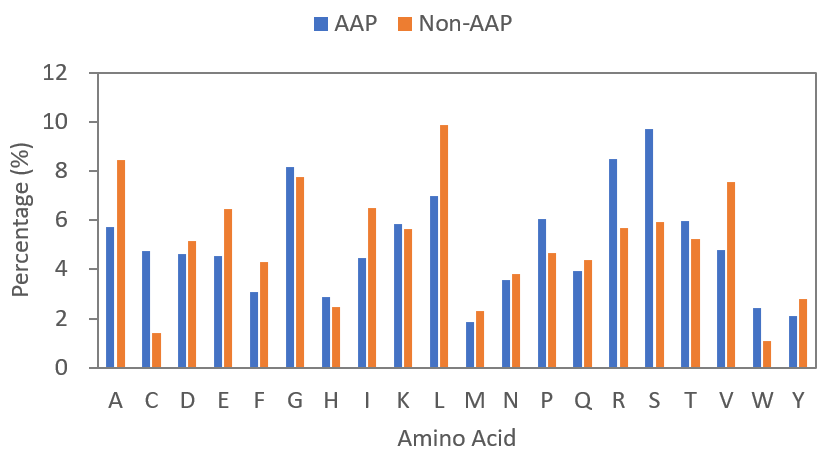


**Figure S1.** Amino acid composition for AAPs and non-AAPs from S212.


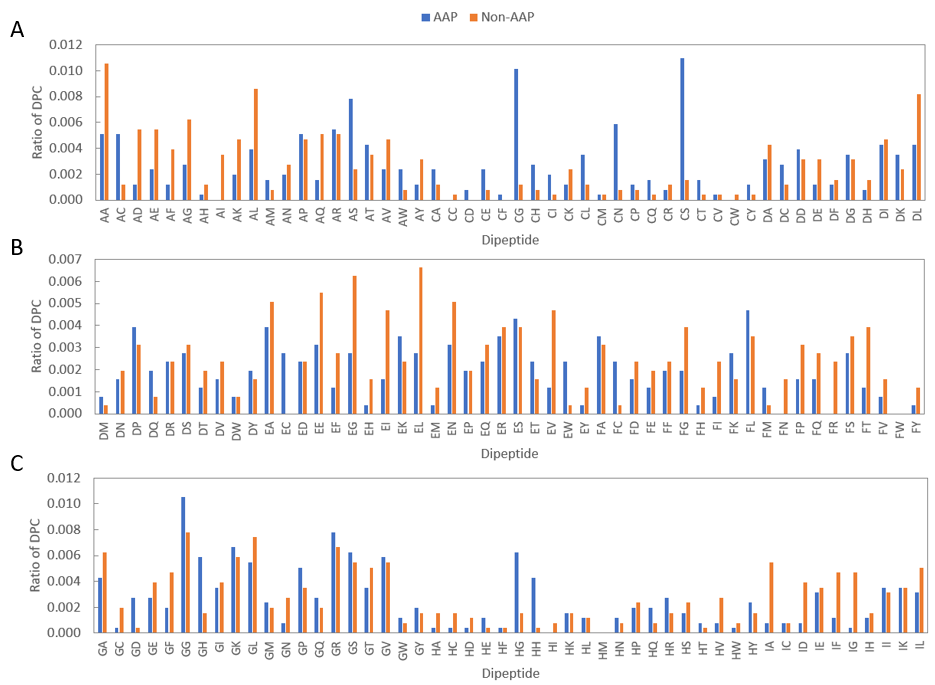


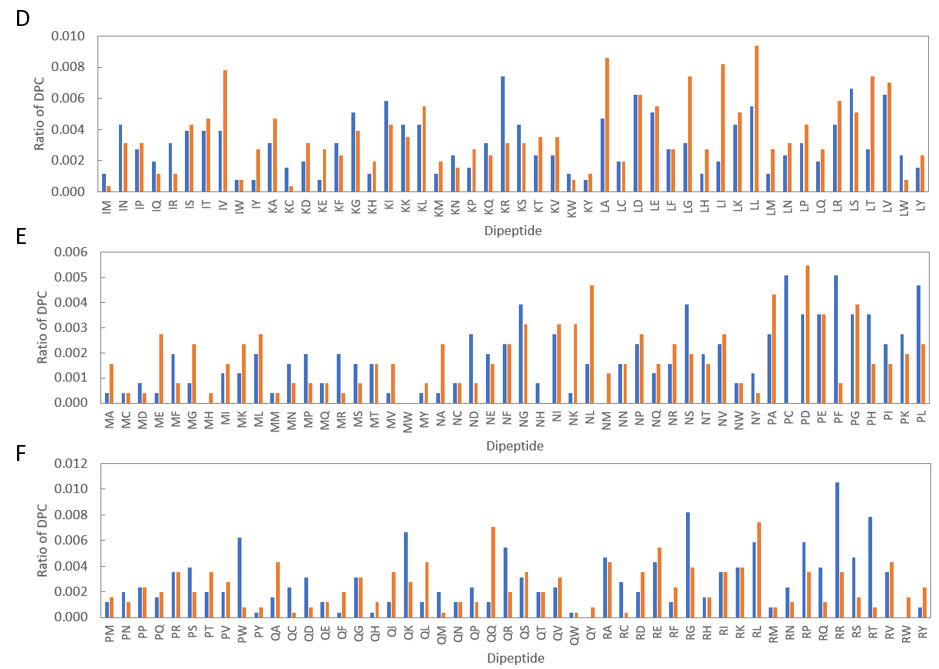


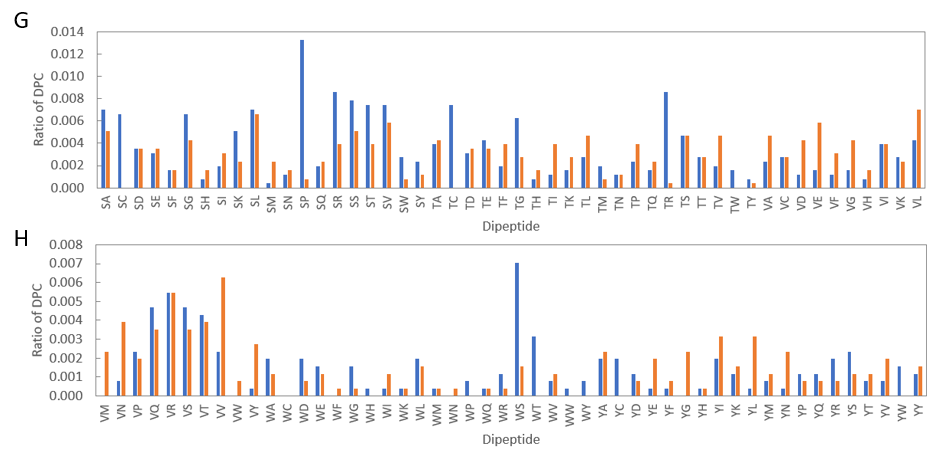


**Figure S2.** Distributions of the 400 dipeptides from AAPs and non-AAPs for S212.


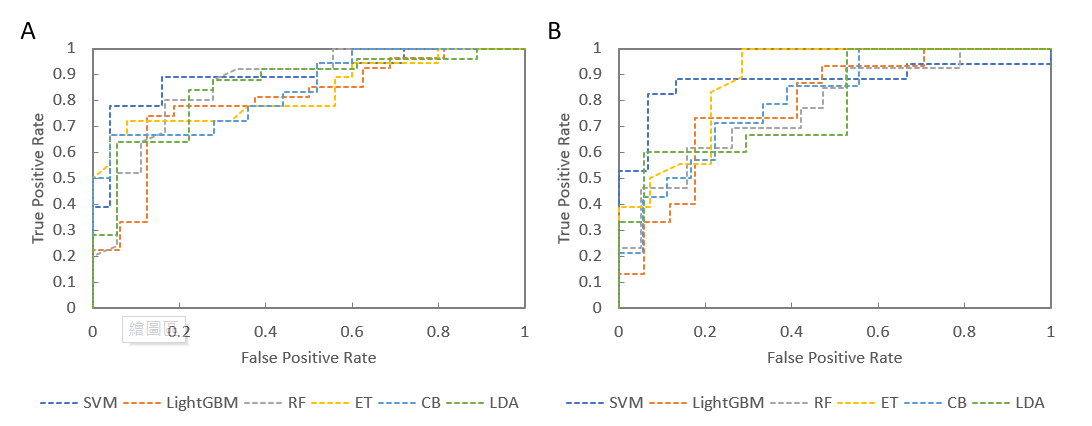


**Figure S3.** ROC curves of the 6 prediction models for A) S212 and B) NT-S160.


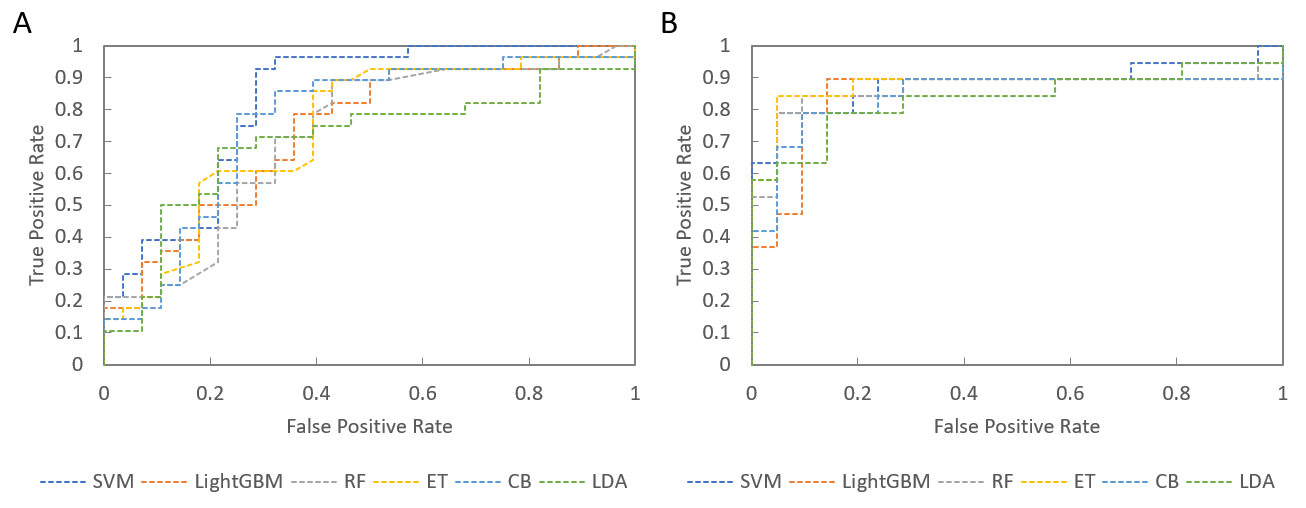


**Figure S4.** ROC curves of the 6 prediction models for A) S56 and B) NT-S40.

**Table S1.** Details of 58 feature types used in the study. I, P, and M in the package column represent iFeature, pFeature, and ModelAMP, respectively.

| Feature Type | Full Name | Size | Package | Ref. |
| --- | --- | --- | --- | --- |
| CKSAAP | Composition of k-spaced Amino Acid Pairs | 1600 | I | [1] |
| DDE | Dipeptide Deviation from Expected mean | 400 | P | [2] |
| DPC | Dipeptide Composition | 400 | P | [3] |
| CTriad | Conjoint Triad | 343 | I | [4] |
| KSCTriad | K-Spaced Conjoint Triad | 343 | I | [5] |
| CTDD | Composition/Transition/**Distribution** | 195 | I | [6] |
| CKSAAGP | Composition of k-Spaced Amino Acid Group Pairs | 150 | I | [5] |
| GTPC | Grouped Tri-Peptide Composition | 125 | I | [7] |
| ABHPRK | Acidic, Basic, Hydrophobic, Polar, aRomatic, Kink-inducer | 105 | M | [8] |
| OVP | Overlapping property | 80 | inhouse | [9] |
| Z5 | The extended five dimensional Z-scale for amino acids | 75 | M | [10] |
| QSO | Quasi-Sequence Order | 46 | P | [11] |
| CTDC | **Composition**/Transition/Distribution | 39 | I | [6] |
| CTDT | Composition/**Transition**/Distribution | 39 | I | [6] |
| Cougar | Modlabs inhouse selection of global peptide descriptors | 30 | M | [8] |
| Ez | Empirical residue-based potential | 30 | M | [12] |
| MSW | The molecular surface based WHIM descriptor | 30 | M | [13] |
| Z3 | The extended three dimensional Z-scale for amino acids | 30 | M | [14] |
| APAAC | Amphiphilic Pseudo-Amino Acid Composition | 26 | I | [15] |
| GDPC | Grouped Di-Peptide Composition | 25 | I | [7] |
| Geary | Geary correlation | 24 | I | [16] |
| Moran | Moran correlation | 24 | I | [17] |
| NMBroto | Normalized Moreau-Broto Autocorrelation | 24 | I | [17] |
| AAC | Amino Acid Composition | 20 | I | [15] |
| DDR | Distance Distribution of Residues | 20 | P | [18] |
| RRI | Repetitive Residue Information | 20 | P | [18] |
| SER | Shannon Entropy at Residue Level | 20 | P | [18] |
| SEP | Shannon Entropy at Protein Level | 20 | P | [18] |
| OVPC | Overlapping property C-terminus | 10 | inhouse | [9] |
| SOCN | Sequence-Order-Coupling Number | 6 | I | [19] |
| GAAC | Grouped Amino Acid Composition | 5 | I | [20] |
| formula | formula | 5 | M | [8] |
| Shannon-Entropy | Shannon Entropy of a property | 1 | P | [18] |
| Length | Length | 1 | M | [21] |
| Calculate_mw | The molecular weight [g/mol] | 1 | M | [22] |
| Calculate_charge | Charge | 1 | M | [23] |
| Isoelectric_point | Isoelectric_point | 1 | M | [21] |
| Instability_index | Instability_index | 1 | M | [24] |
| Aromaticity | Aromaticity | 1 | M | [21] |
| Aliphatic_Index | Aliphatic index | 1 | M | [25] |
| Hydrophobic | Hydrophobic | 1 | M | [21] |
| AASI | Amino acid selectivity index scale | 1 | M | [26] |
| Argos | Argos | 1 | M | [27] |
| Bulkiness | Amino acid side chain bulkiness scale | 1 | M | [28] |
| Charge_phys | Amino acid charge at pH 7. | 1 | M | [29] |
| Charge_acid | Amino acid charge at acidic pH | 1 | M | [29] |
| Flexibility | amino acid side chain flexibilitiy scale | 1 | M | [30] |
| Gravy | GRAVY hydrophobicity amino acid scale | 1 | M | [31] |
| Levitt_alpha | Levitt amino acid alpha-helix propensity scale | 1 | M | [32] |
| MSS | A graph-theoretical index that reflects topological shape and size of amino acid side chains | 1 | M | [33] |
| Polarity | Amino acid polarity scale | 1 | M | [28] |
| Refractivity | Relative amino acid refractivity values | 1 | M | [34] |
| TM_tend | Amino acid transmembrane propensity scale | 1 | M | [35] |
| Boman_Index | Boman index | 1 | M | [36] |
| Eisenberg | The Eisenberg hydrophobicity consensus amino acid scale | 1 | M | [37] |
| Hopp_woods | Hopp-Woods amino acid hydrophobicity scale | 1 | M | [38] |
| Janin | Janin hydrophobicity amino acid scale | 1 | M | [39] |
| Kytedoolittle | Kyte & Doolittle hydrophobicity amino acid scale | 1 | M | [31] |

**References**

[1] K. Chen, L. A. Kurgan, and J. Ruan, “Prediction of protein structural class using novel evolutionary collocation-based sequence representation,” *J. Comput. Chem.*, vol. 29, no. 10, pp. 1596–1604, 2008, doi: 10.1002/jcc.20918.

[2] A. Garg and G. P. S. Raghava, “A Machine Learning Based Method for the Prediction of Secretory Proteins Using Amino Acid Composition, Their Order and Similarity-Search,” *In Silico Biol.*, vol. 8, no. 2, Art. no. 2, Jan. 2008.

[3] A. Garg, M. Bhasin, and G. P. S. Raghava, “Support Vector Machine-based Method for Subcellular Localization of Human Proteins Using Amino Acid Compositions, Their Order, and Similarity Search *,” *J. Biol. Chem.*, vol. 280, no. 15, pp. 14427–14432, Apr. 2005, doi: 10.1074/jbc.M411789200.

[4] J. Shen *et al.*, “Predicting protein–protein interactions based only on sequences information,” *Proc. Natl. Acad. Sci.*, vol. 104, no. 11, pp. 4337–4341, Mar. 2007, doi: 10.1073/pnas.0607879104.

[5] F. Li, X. Guo, D. Xiang, M. E. Pitt, A. Bainomugisa, and L. J. M. Coin, “Computational analysis and prediction of PE_PGRS proteins using machine learning,” *Comput. Struct. Biotechnol. J.*, vol. 20, pp. 662–674, Jan. 2022, doi: 10.1016/j.csbj.2022.01.019.

[6] I. Dubchak, I. Muchnik, S. R. Holbrook, and S. H. Kim, “Prediction of protein folding class using global description of amino acid sequence.,” *Proc. Natl. Acad. Sci. U. S. A.*, vol. 92, no. 19, Art. no. 19, Sep. 1995.

[7] S. Basith, G. Lee, and B. Manavalan, “STALLION: a stacking-based ensemble learning framework for prokaryotic lysine acetylation site prediction,” *Brief. Bioinform.*, vol. 23, no. 1, Art. no. 1, Jan. 2022, doi: 10.1093/bib/bbab376.

[8] P. B. Timmons and C. M. Hewage, “ENNAACT is a novel tool which employs neural networks for anticancer activity classification for therapeutic peptides,” *Biomed. Pharmacother.*, vol. 133, p. 111051, Jan. 2021, doi: 10.1016/j.biopha.2020.111051.

[9] B. Manavalan, S. Basith, T. H. Shin, L. Wei, and G. Lee, “mAHTPred: a sequence-based meta-predictor for improving the prediction of anti-hypertensive peptides using effective feature representation,” *Bioinformatics*, vol. 35, no. 16, pp. 2757–2765, Aug. 2019, doi: 10.1093/bioinformatics/bty1047.

[10] M. Sandberg, L. Eriksson, J. Jonsson, M. Sjöström, and S. Wold, “New Chemical Descriptors Relevant for the Design of Biologically Active Peptides. A Multivariate Characterization of 87 Amino Acids,” *J. Med. Chem.*, vol. 41, no. 14, Art. no. 14, Jul. 1998, doi: 10.1021/jm9700575.

[11] K.-C. Chou, “Prediction of Protein Subcellular Locations by Incorporating Quasi-Sequence-Order Effect,” *Biochem. Biophys. Res. Commun.*, vol. 278, no. 2, Art. no. 2, Nov. 2000, doi: 10.1006/bbrc.2000.3815.

[12] A. Senes, D. C. Chadi, P. B. Law, R. F. S. Walters, V. Nanda, and W. F. DeGrado, “Ez, a Depth-dependent Potential for Assessing the Energies of Insertion of Amino Acid Side-chains into Membranes: Derivation and Applications to Determining the Orientation of Transmembrane and Interfacial Helices,” *J. Mol. Biol.*, vol. 366, no. 2, pp. 436–448, Feb. 2007, doi: 10.1016/j.jmb.2006.09.020.

[13] A. Zaliani and E. Gancia, “MS-WHIM Scores for Amino Acids:  A New 3D-Description for Peptide QSAR and QSPR Studies,” *J. Chem. Inf. Comput. Sci.*, vol. 39, no. 3, pp. 525–533, May 1999, doi: 10.1021/ci980211b.

[14] S. Hellberg, M. Sjoestroem, B. Skagerberg, and S. Wold, “Peptide quantitative structure-activity relationships, a multivariate approach,” *J. Med. Chem.*, vol. 30, no. 7, pp. 1126–1135, Jul. 1987, doi: 10.1021/jm00390a003.

[15] K.-C. Chou, “Prediction of protein cellular attributes using pseudo-amino acid composition,” *Proteins Struct. Funct. Bioinforma.*, vol. 43, no. 3, pp. 246–255, 2001, doi: 10.1002/prot.1035.

[16] R. R. Sokal and B. A. Thomson, “Population structure inferred by local spatial autocorrelation: An example from an Amerindian tribal population,” *Am. J. Phys. Anthropol.*, vol. 129, no. 1, pp. 121–131, 2006, doi: 10.1002/ajpa.20250.

[17] D. S. Horne, “Prediction of protein helix content from an autocorrelation analysis of sequence hydrophobicities,” *Biopolymers*, vol. 27, no. 3, pp. 451–477, Mar. 1988, doi: 10.1002/bip.360270308.

[18] A. Pande *et al.*, “Pfeature: A Tool for Computing Wide Range of Protein Features and Building Prediction Models,” *J. Comput. Biol.*, vol. 30, no. 2, pp. 204–222, Feb. 2023, doi: 10.1089/cmb.2022.0241.

[19] Z. Chen *et al.*, “iFeature: a Python package and web server for features extraction and selection from protein and peptide sequences,” *Bioinformatics*, vol. 34, no. 14, pp. 2499–2502, Jul. 2018, doi: 10.1093/bioinformatics/bty140.

[20] T.-Y. Lee, Z.-Q. Lin, S.-J. Hsieh, N. A. Bretaña, and C.-T. Lu, “Exploiting maximal dependence decomposition to identify conserved motifs from a group of aligned signal sequences,” *Bioinformatics*, vol. 27, no. 13, pp. 1780–1787, Jul. 2011, doi: 10.1093/bioinformatics/btr291.

[21] A. J. Velez Rueda, F. L. Bulgarelli, N. Palopoli, and G. Parisi, “CaviDB: a database of cavities and their features in the structural and conformational space of proteins,” *Database*, vol. 2023, p. baad010, Jan. 2023, doi: 10.1093/database/baad010.

[22] S. N. Dean and S. A. Walper, “Variational Autoencoder for Generation of Antimicrobial Peptides,” *ACS Omega*, vol. 5, no. 33, pp. 20746–20754, Aug. 2020, doi: 10.1021/acsomega.0c00442.

[23] J. M. Heather *et al.*, “Murine xenograft bioreactors for human immunopeptidome discovery,” *Sci. Rep.*, vol. 9, no. 1, Art. no. 1, Dec. 2019, doi: 10.1038/s41598-019-54700-2.

[24] K. Guruprasad, B. V. B. Reddy, and M. W. Pandit, “Correlation between stability of a protein and its dipeptide composition: a novel approach for predicting in vivo stability of a protein from its primary sequence,” *Protein Eng. Des. Sel.*, vol. 4, no. 2, Art. no. 2, Dec. 1990, doi: 10.1093/protein/4.2.155.

[25] A. Ikai, “Thermostability and Aliphatic Index of Globular Proteins,” *J. Biochem. (Tokyo)*, vol. 88, no. 6, pp. 1895–1898, 1980.

[26] “Computational Design of Highly Selective Antimicrobial Peptides | Journal of Chemical Information and Modeling.” Accessed: Jun. 26, 2023. [Online]. Available: https://pubs.acs.org/doi/10.1021/ci900327a

[27] P. Argos, J. K. M. Rao, and P. A. Hargrave, “Structural Prediction of Membrane-Bound Proteins,” *Eur. J. Biochem.*, vol. 128, no. 2–3, Art. no. 2–3, 1982, doi: 10.1111/j.1432-1033.1982.tb07002.x.

[28] J. M. Zimmerman, N. Eliezer, and R. Simha, “The characterization of amino acid sequences in proteins by statistical methods,” *J. Theor. Biol.*, vol. 21, no. 2, Art. no. 2, Nov. 1968, doi: 10.1016/0022-5193(68)90069-6.

[29] P. J. A. Cock *et al.*, “Biopython: freely available Python tools for computational molecular biology and bioinformatics,” *Bioinformatics*, vol. 25, no. 11, Art. no. 11, Jun. 2009, doi: 10.1093/bioinformatics/btp163.

[30] R. Bhaskaran and P. k. Ponnuswamy, “Positional flexibilities of amino acid residues in globular proteins,” *Int. J. Pept. Protein Res.*, vol. 32, no. 4, Art. no. 4, 1988, doi: 10.1111/j.1399-3011.1988.tb01258.x.

[31] J. Kyte and R. F. Doolittle, “A simple method for displaying the hydropathic character of a protein,” *J. Mol. Biol.*, vol. 157, no. 1, Art. no. 1, May 1982, doi: 10.1016/0022-2836(82)90515-0.

[32] M. Levitt, “Conformational preferences of amino acids in globular proteins,” *Biochemistry*, vol. 17, no. 20, pp. 4277–4285, Oct. 1978, doi: 10.1021/bi00613a026.

[33] C. Raychaudhury, A. Banerjee, P. Bag, and S. Roy, “Topological Shape and Size of Peptides:  Identification of Potential Allele Specific Helper T Cell Antigenic Sites,” *J. Chem. Inf. Comput. Sci.*, vol. 39, no. 2, Art. no. 2, Mar. 1999, doi: 10.1021/ci980052w.

[34] T. L. McMeekin, M. Wilensky, and M. L. Groves, “Refractive indices of proteins in relation to amino acid composition and specific volume,” *Biochem. Biophys. Res. Commun.*, vol. 7, no. 2, Art. no. 2, Apr. 1962, doi: 10.1016/0006-291X(62)90165-1.

[35] G. Zhao and E. London, “An amino acid ‘transmembrane tendency’ scale that approaches the theoretical limit to accuracy for prediction of transmembrane helices: Relationship to biological hydrophobicity,” *Protein Sci.*, vol. 15, no. 8, Art. no. 8, 2006, doi: 10.1110/ps.062286306.

[36] H. G. Boman, D. Wade, I. A. Boman, B. Wåhlin, and R. B. Merrifield, “Antibacterial and antimalarial properties of peptides that are cecropin-melittin hybrids,” *FEBS Lett.*, vol. 259, no. 1, pp. 103–106, Dec. 1989, doi: 10.1016/0014-5793(89)81505-4.

[37] D. Eisenberg, R. M. Weiss, T. C. Terwilliger, and W. Wilcox, “Hydrophobic moments and protein structure,” *Faraday Symp. Chem. Soc.*, vol. 17, no. 0, Art. no. 0, Jan. 1982, doi: 10.1039/FS9821700109.

[38] T. P. Hopp and K. R. Woods, “Prediction of protein antigenic determinants from amino acid sequences.,” *Proc. Natl. Acad. Sci.*, vol. 78, no. 6, Art. no. 6, Jun. 1981, doi: 10.1073/pnas.78.6.3824.

[39] J. L. Cornette, K. B. Cease, H. Margalit, J. L. Spouge, J. A. Berzofsky, and C. DeLisi, “Hydrophobicity scales and computational techniques for detecting amphipathic structures in proteins,” *J. Mol. Biol.*, vol. 195, no. 3, pp. 659–685, Jun. 1987, doi: 10.1016/0022-2836(87)90189-6.
